# Differences in sprinting performance and kinematics between preadolescent boys who are fore/mid and rear foot strikers

**DOI:** 10.1371/journal.pone.0205906

**Published:** 2018-10-18

**Authors:** Aya Miyamoto, Tomonari Takeshita, Toshio Yanagiya

**Affiliations:** Graduate School of Health and Sports Science, Juntendo University, Inzai-City, Chiba, Japan; Universite de Nantes, FRANCE

## Abstract

The purpose of this study was to clarify whether foot strike patterns are associated with different sprint performance and kinematics in preadolescent boys. The study enrolled 24 healthy 10–11-year-old boys in the fifth grade at public elementary schools in Japan. The participants performed the 50-m sprint with maximum effort. Sprint motion was recorded using a high-speed video camera (120 fps) placed in the sagittal plane on the left side of a line drawn at 35-m from the start line. Kinematic variables were calculated based on manually digitized body landmark coordinates. The participants were categorized into two groups according to their foot strike pattern (rearfoot strikers, RF group, n = 12; forefoot or midfoot strikers, FF/MF group, n = 12). The time taken to complete the 50-m sprint in the FF/MF group (9.08±0.52 s) was faster than that in the RF group (9.63±0.51 s). The FF/MF group had greater sprint speed, higher step frequency, and shorter foot contact time than the RF group. Regarding the association between foot strike pattern and sprint kinematics, we found that the RF group had a greater range of knee flexion during the support-leg phase, whereas the FF/MF group had shorter horizontal distance from the heel of the support leg to the centre of mass at the touchdown, greater maximal knee flexion velocity during the swing-leg phase, and higher the maximum hip extension velocity during the support-leg phase. The current results suggested that, in preadolescent boys, forefoot or midfoot strike (rather than rearfoot strike) is effective for obtaining a higher step frequency and sprint speed through greater magnitude of knee flexion and hip extension movement velocities during the swing and support phases, respectively. The current findings will be useful for understanding the characteristics of the development of sprinting performance in preadolescent children.

## Introduction

Sprinting and sub-maximal running are key fundamental exercises that are considered to be the basis for many sporting activities. Furthermore, sprint tests are used extensively as an evaluation of growth or talent, with maximum sprint speed as a measurable indicator of performance [1–4]. Sprint speed represents by the product of step length and step frequency. Thus, an increase in either of the two factors results in improved sprint speed, provided that the other factor remains unchanged or does not decrease substantially [5].

Previous studies have indicated that development of sprint performance with growth in childhood has a major impact on the increase in step length, which accompanies morphological development [2–4]. Meanwhile, step frequency does not change or may even decline with growth [2–4]. However, it has been verified that maximum sprint speed has a significant positive correlation with both step length and step frequency in 11–15-year-old boys [2]. In addition, it was reported that both step length and step frequency are important factors to explain individual differences in maximum sprint speed among Japanese elementary school students in the same age group [4]. Accordingly, the better sprint performance of preadolescent children is characterized not only by longer step length but also by higher step frequency.

Foot strike pattern is one factor that relates to magnitudes of step length and frequency [6, 7]. Foot strike patterns are classified into three types, according to the position of the foot upon landing; these include the forefoot strike (FF), midfoot strike (MF), and rearfoot strike (RF) [7]. Foot strike patterns can also be classified as heel or non-heel contact. Based on the studies employed adult runners, FF is characterized by high step frequency and short step length [6–8]. Additionally, adult FF and MF runners have shorter foot contact times compared with adult RF runners [8–10]. Furthermore, kinematic and kinetic characteristics also differ according to foot strike pattern, with RF known to cause large impact and loading rate at touchdown [6–14]. Because the basis of locomotion is same between children and adults, the foot strike pattern would relate to spatiotemporal, kinematic and kinetic variables of sprinting in children. However, since previous studies on foot strike kinematics focused on only adult participants, such aspects have not been clarified in children. As an another perspective, previous studies have reported that, as a result of natural development, boys at pre-peak height velocity (PHV) showed decrease in step frequency and increase in foot contact time [2]. These changes have previously been explained as temporary disruption of motor coordination, often termed "adolescent awkwardness" [2, 3]. Investigating the association of foot strike patterns with spatiotemporal and kinematic variables at the age of "adolescent awkwardness" might bring the insight of specific feature of development of sprinting performance. The purpose of the present study was to clarify whether foot strike patterns are associated with different sprint performance and kinematics in preadolescent boys. The findings of this study are expected to be useful in understanding of the characteristics of the development of sprinting performance in preadolescent children.

## Methods

### Participants

Twenty-four healthy boys enrolled in the fifth grade at two public elementary schools in Japan (10–11 years old) participated in this study (body height, 1.40 ± 0.07 m; body mass, 35.5 ± 6.9 kg). This study adopted the body height of 1.54 m as the threshold indicative of adolescence, based on body height data reported for Japanese children at PHV, and in a development stage corresponding to adolescence on the Tanner’s index [15]. For all participants, body height at the time of the study was <1.54m, indicating that all participants were pre-PHV, preadolescent children.

Prior to the study, the participants and their guardians received a verbal and written explanation of the objective and methods of the study. Written informed consent was obtained from the guardians, and assent was obtained from the participants. In addition, various cautionary instructions were verbally communicated to the participants throughout the study. This study was conducted in accordance with the Declaration of Helsinki and was approved by the Research Ethics Committee of the Graduate School of Health and Sports Science, Juntendo University (approval number 27–112 at Graduate School of Juntendo University).

### Data collection

All measurements were conducted during the regular a physical education class at the elementary school. The experiment consisted of a single, timed 50-m sprint test conducted independently for each participant. Each participant started from a stationary standing position; upon receiving a start cue from the examiner (who exclaimed "Go!" and swung a flag from up to down), the participant ran with maximum effort for 50-m from the starting line, and the total time was recorded. Prior to the start of the experiment itself, we conducted a pilot study to assess the variability of measurements of time for the 50-m sprint test among male elementary school children in fifth grade (n = 14). We confirmed that variability among individuals was below 5%, with an intraclass correlation coefficient of 0.932 (p<0.01).

The 50-m sprint test was carried out on a straight gravel track that served as a runway. Such tracks are commonly used for physical education activities in elementary schools in Japan. The participants wore the shoes they usually uses for physical education lessons. Prior to the sprint trial, the participants performed warm-up exercises, including running and sprint running in addition to the regular preparatory exercises performed routinely during physical education classes.

A video camera (GC-PX 1, 60 fps; JVC KENWOOD Corporation, Kanagawa, Japan) was set in the sagittal plane, on the left side of the finish line (distance from the runway, 15 m; height, 0.8 m). The recorded video was used to accurately determine the total time between the moment the start cue was issued and the moment when the participant crossed the finish line situated at 50-m from the start line. In addition, a high-speed digital video camera (EXILIM EX-FH25; CASIO COMPUTER Co., Ltd, Tokyo, Japan) was set in the sagittal plane on the left side of a line drawn at a distance of 35 m from the start line (distance from the runway, 19.6 m; height, 0.8 m) to record the sprint motion (frame rate, 120 fps; shutter speed, 1/1250s; resolution, 640 × 480 pixels; light sensitivity, ISO 400; aperture, F 3.7; focal length, 82 mm). The high-speed camera was fixed onto a tripod so that the optical axis was perpendicular to the runway. The field of view spanned a total of 6 m, covering 3 m before and after the line situated at 35-m from the starting line.

### Data analysis

Using the images captured by the video camera set at the finish line, the time of the 50-m sprint test was calculated based on the number of frames between the frame containing the start cue and the frame showing the participant crossing the finish line. The starter cue consisted of the examiner exclaiming "Go!" and swing a flag from up to down. The moment at which the flag began to move downwards was considered the start time.

Participants were categorized into two groups according to their foot strike pattern, which was determined based on the images recorded by the high-speed camera (S1 Fig). Specifically, playback software (Quick Time Player, Version 7; Apple Inc., Cupertino, CA) was used to advance through the video of each participant frame-by-frame and determine which part of the foot was the first to touch the ground. RF pattern was noted for 12 participants, who were thus included in RF group, while FF or MF pattern was noted for the other 12 participants, who were thus included in FF/MF group [13].

The images recorded by the high-speed video camera were also used to determine step frequency, foot contact time, and aerial time. First, stride time (in units of s) was defined as the time between two consecutive touchdowns of the left foot. Since the stride covers two steps, step frequency (in units of steps/s) was then calculated as 2 / stride time. Foot contact time was defined as the time from touchdown to takeoff of the left foot. Aerial time was defined as the time from takeoff of the left foot to touchdown of the right foot.

Using a kinematic analysis software (Frame-DIAS IV; DKH Inc., Tokyo, Japan), 23 body landmarks (vertex, suprasternale, midpoint between both tragious, shoulder joint centres, elbow joint centres, wrist joint centres 3^rd^ metacarpophalangeal joints, hip joint centres, knee joint centeres, ankle joint centers, heels, metatarsophalangeal joints, and toes) were manually digitized in the images recorded by the high-speed camera (120 Hz). Then, the two-dimensional coordinates (x and y) were extracted. The body landmark were chosen based on the body segment model defined in Japanese children [16]. For each stride, this manual digitizing started at 10 frames before touchdown of the left foot and ended at 10 frames after the subsequent touchdown of the same foot. Calibration was performed according to the 2D-4Points method implemented in the kinematic analysis software, which employed the position of calibration markers placed along each side of the runway (every 6 m along the x-axis, 1 m in depth; S1 Fig) to interpolate the position of each body landmark in the 2D space. The aspect ratio of the image was measured by placing square frame (1 m × 1 m) at the centre of the image. The 2D position data were smoothed using a Butterworth digital low-pass filter with a cut-off frequency of 10 Hz. To evaluate the reliability of 2D measurements via this method, the same researcher repeated 10 times a sprint motion analysis of a participant. The coefficient of variation for data of 10 times was below 3%.

Sprint motion was divided into two phases, namely the support-leg and the swing-leg phase, and the following kinematic variables describing leg motion were obtained (S2 Fig). Extension or flexion velocity of each joint was calculated as the rate of change of angular displacement in time. The joint range of motion (ROM) was calculated between the joint angle value at touchdown and the minimum joint value (flexion), or between the minimum value and the value noted at takeoff (extension).

Centre-of-mass (COM) position data were obtained from the digitized 2D data represented using the rigid-segment body model proposed for Japanese children aged 9–11 years old [16]. Step length was calculated as half distance of horizontal displacement of COM from touchdown of the left leg to the next touchdown of the left leg (since there were two step per stride). Sprint speed was calculated as the product of the step length and frequency. The COM heights at the lowest and highest points, and at touchdown and takeoff were obtained. Vertical displacement of COM was calculated as the difference in heights between the lowest and highest position. Horizontal displacement of COM was measured for the support (as the distance from touchdown to takeoff of the left leg) and aerial phases (as the distance from takeoff of the left leg to touchdown of the right leg). Furthermore, the horizontal distance from the left heel to the COM at touchdown was determined.

### Statistical analyses

All calculated variables were expressed as mean ± standard deviation of the distribution noted in each group. The significance of between-group differences was tested using the unpaired t-test. In addition, an analysis of covariance (ANCOVA) was used to compare differences in the spatiotemporal variables between the two groups with sprint speed as the covariate. The Pearson’s correlation coefficient was determined for the correlation among spatiotemporal variables. In addition, the correlations were calculated between sprint speed and kinematics variables. For all tests, the significance level was set at p < 0.05. All statistical analyses were performed using statistical analysis software (SPSS Statistics, version 22.0; IBM Corp., Armonk, NY).

## Results

The physical characteristics of participants included in each groups are summarized in Table 1. There was no significant difference between the RF and FF/MF groups with regard to physical characteristics.

**Table 1:** Body height and body mass statistics of the study participants.

Characteristic	RF group(n = 12)	FF/MF group (n = 12)	*p*
Body height (m)	1.42 ± 0.06	1.38 ± 0.08	0.242
Body mass (kg)	37.8 ± 7.4	33.1 ± 5.6	0.094

The results of the 50-m sprint (time, sprint speed, and spatiotemporal variables) are summarized in Table 2. Based on the sprint time, the FF/MF group was significantly faster than RF group (p = 0.017). Additionally, sprint speed was significantly higher in the FF/MF group than in the RF group, in agreement with the trend noted for step frequency, which was also significantly higher in the FF/MF group.

**Table 2:** Descriptive data regarding sprint performance in the 50-m sprint test.

Variable	RF group (n = 12)	FF/MF group (n = 12)	*p*
Time on the 50-m sprint test (s)	9.63 ± 0.51	9.08 ± 0.52	0.017
Sprint speed (m/s)	6.08 ± 0.40	6.53 ± 0.46	0.017
Step length (m)	1.58 ± 0.14	1.53 ± 0.11	0.303
Step frequency (step/s)	3.86 ± 0.22	4.29 ± 0.28	0.001
Foot contact time (s)	0.160 ± 0.013	0.132 ± 0.013	0.001
Aerial time (s)	0.108 ± 0.022	0.099 ± 0.012	0.180

The *p* value shows the result of the t-test.

The results of Pearson’s correlation matrix are shown in Table 3. There were significant correlations of sprint speed with step length, step frequency, and foot contact time. The step length was significantly correlated with step frequency.

**Table 3:** Pearson’s correlation matrix among spatiotemporal variables.

Variable		Sprint speed	Step length	Step frequency	Foot contact time	Aerial time
Time on the 50-m sprint test	r	-.966	-.446	-.493	.607	0.089
	*p*	0.001	0.029	0.014	0.002	0.679
Sprint speed	r		.460	.507	-.603	-.085
	*p*		0.024	0.011	0.002	0.693
Step length	r			-.529	.139	.595
	*p*			0.008	0.518	0.002
Step frequency	r				-.705	-.662
	*p*				0.001	0.001
Foot contact time	r					.112
	*p*					0.601

Table 4 shows the results of ANCOVA. There was no interaction between the covariate and any of the variables. There were significant differences between the groups regarding step length, step frequency, and foot contact time (p<0.01).

**Table 4:** Results of ANCOVA in the spatiotemporal variables between the groups with sprint speed as the covariate.

Source		Sum of Squares	df	Mean Square	F	*p*
Sprint speed	Step length	0.143	1	0.143	16.394	0.001
	Step frequency	0.115	1	0.115	1.879	0.185
	Foot contact time	0.001	1	0.001	4.444	0.047
	Aerial time	2.516E-05	1	2.516E-05	0.080	0.780
Group	Step length	0.087	1	0.087	9.957	0.005
	Step frequency	0.569	1	0.569	9.272	0.006
	Foot contact time	0.002	1	0.002	15.556	0.001
	Aerial time	0.001	1	0.001	1.756	0.199
Error	Step length	0.184	21	0.009		
	Step frequency	1.288	21	0.061		
	Foot contact time	0.003	21	0.000		
	Aerial time	0.007	21	0.000		

Table 5 summarizes the data regarding the kinematic variables describing sprint motion in each group. In the support-leg phase, there was a significant difference between the two groups (p = 0.017) regarding the ROM of knee flexion, which was more ample in the RF group. The maximum hip extension velocity was higher in the FF/MF group. In the swing-leg phase, the maximal knee flexion velocity was significantly greater in the FF/MF group than in the RF group (p = 0.048). The RF group showed significantly greater values of COM vertical and horizontal displacements in the support-leg phase, and the horizontal distance from the heel to the COM at touchdown compared to the FF/MF group.

**Table 5:** Descriptive data for each group in kinematics variables.

Variable		RF group (n = 12)	FF/MF group (n = 12)	*p*
Support-leg phase				
Joint angle at touchdown (degree)	Hip	116.7 ± 7.9	118.1 ± 8.9	0.673
	Knee	142.0 ± 3.9	139.3 ± 7.2	0.354
	Ankle	120.9 ± 7.6	126.4 ± 8.8	0.115
Minimum joint angle (degree)	Hip	116.7 ± 7.9	118.1 ± 8.9	0.673
	Knee	123.5 ± 7.4	125.3 ± 7.3	0.566
	Ankle	108.0 ± 9.6	115.8 ± 9.5	0.057
Joint angle at takeoff (degree)	Hip	175.4 ± 3.5	175.2 ± 4.0	0.887
	Knee	147.7 ± 5.6	148.5 ± 6.2	0.770
	Ankle	147.7 ± 7.8	151.1 ± 6.0	0.247
ROM	Hip extension	51.9 ± 6.3	50.0 ± 7.8	0.505
	Knee flexion	-18.5 ± 4.2	-14.1 ± 4.2	0.017
	Knee extension	24.2 ± 4.3	23.2 ± 4.7	0.578
	Ankle flexion	-13.0 ± 6.5	-10.6 ± 5.8	0.364
	Ankle extension	39.7 ± 6.4	35.3 ± 7.8	0.136
Maximum extension velocity (degree/s)	Hip	686.7 ± 115.7	793.9 ± 98.1	0.023
	Knee	490.1 ± 83.3	536.4 ± 85.0	0.191
	Ankle	707.8 ± 145.4	707.9 ± 149.8	0.999
Swing-leg phase				
Maximum hip flexion angle (degree)		57.9 ± 5.8	61.6 ± 6.2	0.151
Maximum hip flexion velocity (degree/s)		616.9 ± 84.3	672.9 ± 75.0	0.100
Minimum knee flexion angle (degree)		41.8 ± 13.5	39.3 ± 12.4	0.644
Maximum knee flexion velocity (degree/s)		-932.4 ± 112.3	-1026.0 ± 106.8	0.048
COM trajectory				
The COM height (cm)	At lowest point	79.6 ± 4.0	77.1 ± 4.6	0.170
	At highest point	86.0 ± 4.5	82.3 ± 4.8	0.068
	At touchdown	81.9 ± 4.4	78.9 ± 4.8	0.122
	At takeoff	85.2 ± 4.2	81.7 ± 4.8	0.072
Vertical displacement (cm)		6.4 ± 1.2	5.2 ± 1.0	0.017
Horizontal displacement (cm)	For the Support	95.6 ± 8.0	85.7 ± 7.5	0.005
	For the Aerial	67.2 ± 13.8	66.4 ± 9.8	0.873
Horizontal distance from the heel to the COM at touchdown (cm)		15.3 ± 3.8	9.3 ± 3.1	0.001

The *p* value shows the result of the t-test.

As results of correlation test of sprint speed with kinematics, the maximum support hip extension velocity (r = 0.504, p = 0.012) was significantly and positively correlated with sprint speed. The maximum swing hip flexion angle (r = 0.513, p = 0.010) and maximum swing hip flexion velocity (r = 0.408, p = 0.048) were significantly and positively correlated with sprint speed. Moreover, there was significant and negative correlation between the maximum knee flexion velocity and sprint speed (r = -0.435, *p* = 0.034).

## Discussion

The aim of this study was to clarify whether foot strike patterns are associated with different sprint performance and kinematics in preadolescent boys. The results of this study demonstrate that there were indeed significant differences in sprint performance and kinematics between RF and non-RF groups. Firstly, we found that sprint speed (reflected also as the time needed to complete the 50-m sprint test) was higher in children with FF or MF than in those with RF, suggesting that FF and MF is likely associated with better sprinting performance. Secondly, we found that the RF had a greater range of knee flexion during the support-leg phase, whereas the FF or MF had shorter horizontal distance from the heel of the support leg to the centre of mass at the touchdown, greater maximal knee flexion velocity during the swing-leg phase, and higher the maximum hip extension velocity during the support-leg phase.

Previous study revealed that male and female 100-m sprinters participating in the Olympics all sprinted with FF [17]. Moreover, studies evaluated the foot strike patterns and the finish times in middle-distance and/or long-distance races have found that the mean speed in 800-m or 1500-m races was higher among FF and MF runners than among RF runners [9]. Although some other studies have found no difference in running performance associated with different foot strike patterns [8, 18], greater sprinting performance in FF/MF group in the present study is in line with the aforementioned previous studies [9, 17].

It is currently recognized that running speed affects foot strike pattern [19–21]. Studies regarding changes in foot strike patterns that accompany an increase in within-person running speeds reported that, when running speed exceeded 5 m/s, there was a shift toward more anterior strike patterns (i.e., away from RF and toward MF and FF patterns) [20], and this shift occurred in approximately half of the runners [19]. The influence of the running speed on the foot strike pattern is also reflected in the proportion of RF runners participating in events requiring different speeds. Whereas all of male and female 100-m sprinters participating in the Olympics showed FF [17], 27% of runners in 800-m and 1500-m races and 74.9% of runners in half marathon adopted RF [8,9]. These previous findings indicated that running speed is inversely associated with the prevalence of RF. Consequently, there is a possibility that the increment of running speed due to natural growth resulted in the changes in foot strike pattern from RF to FF/MF, and thus the difference in the sprint performance associated with the difference in foot strike pattern would be led by the difference in maturity level.

Our finding showed that the foot contact time was shorter in the FF/MF group than in the RF group. This finding is agreement with previous findings that FF or MF was associated with shorter foot contact time [8–10]. However, sprint speed was significantly higher in the FF/MF group than in the RF group in this study, which should be discussed in the context of spatiotemporal variables. This study showed significantly correlation of sprint speed with step length, step frequency, and foot contact time. The ANCOVA results confirmed that the step length (shorter in FF/MF group), step frequency (higher in FF/MF group) and contact time (shorter in FF/MF group) were different due to foot strike pattern regardless of sprint speed. These results were similar to previous studies [6–8] and it can be said that FF and MF are characterized by high step frequency and short step length. Additionally, in terms of spatiotemporal variables and sprint motion, preadolescent boys with RF may reflect the characteristics of boys with “adolescent awkwardness” [2, 3]. A previous study reported that the pre-PHV period is characterized by increased foot contact time and decreased step frequency, potentially resulting from challenges associated with differential growth patterns; the authors pointed out that the pre-PHV period is thus a key time window, in which training should focus upon technical drills to offset this growth-related decline in sprint performance [22]. Taken together, the current findings are likely useful to consider training program in preadolescent boys for improving sprinting performance through changes in the foot strike pattern from RF to MF or FF.

Regarding the effect of foot strike pattern on sprint kinematics, several aspects should be discussed. The ROM of knee flexion during the support-leg phase was greater in the RF group than in the FF/MF group, which is in line with previous findings in adults [11, 14, 23]. Moreover, compared to the RF group, the FF/MF group had shorter horizontal distance from the heel of the support side to the COM at touchdown, greater maximum knee flexion velocity during the swing-leg phase, and higher maximum hip extension velocity during the support-leg phase. These findings might be explained by previous observations that, in FF strikers, loading typically occurs directly under the COM [6, 10, 12]. Furthermore, there were significant correlation of sprint speed with variables, namely the maximum knee flexion velocity during the swing-leg phase and the maximum hip extension velocity during the support-leg phase. These findings indicate that the specific characteristics of sprint kinematics in the FF/FM group shown here were related to the higher sprint speed.

Our present results confirm that the effect of foot strike pattern on sprint kinematics is similar between children and adults [6–14, 23]. In addition, it is interesting that the differences in sprint motion manifest not only during the support-leg phase but also during the swing-leg phase. De Wit et al. [6] showed that barefoot running induced flatter foot placement at touchdown, which was facilitated by pre-conditioning of the lower limb well before touchdown; such pre-conditioning included larger plantar flexion, more knee flexion, and a large knee flexion velocity. Moreover, Ahn et al. [13] explained that, compared to RF runners, FF runners activated their plantar flexor muscles earlier and for longer not only in preparation for landing but also as a mechanism to increase the capacity of passive structures to store elastic energy at the beginning of stance. There is a possibility that the foot strike pattern may be influence by the difference of motion at the landing preparation, so further analysis is necessary in the future.

This study has several limitations and strengths that warrant discussion. One limitation is related to the fact that the experiments were conducted during the physical education classes at elementary schools. Thus, the collected data are expected to have lower precision than would be obtained under laboratory conditions because we could not use equipment typically employed, such as 3-D motion capture technology. Moreover, although the use of motion markers can provide better tracking and digitization of body landmark coordinates, we refrained from sticking markers onto the skin of the children as that would have hindered their running and would have required substantial disruption of the physical education classes. On the other hand, conducting the experiments as part of the physical education class allowed us to collect data pertaining to the general population of school children, regardless of athletic ability. We hope to be able to recreate such experiments in the near future, using more accurate measurements equipment. Another limitation of this study is that foot strike was evaluated for a single step in the 50-m sprint. Therefore, we could not check how many steps were made and how step length and frequency changed over the entire distance (50 m). However, this study was the first to clarify the differences in sprint speed and sprint motion associated with different foot strike patterns in children. This information may be used to plan future studies in children.

## Conclusions

We found that foot strike patterns were associated with different sprint performance and kinematics in preadolescent boys. Specifically, sprint speed was higher and 50-m sprint time was shorter in FF and MF strikers than RF strikers. Moreover, FF and MF strikers were associated with higher step frequency and shorter foot contact time. Regarding kinematics, RF strikers were associated with greater ROM of knee flexion during the support-leg phase, whereas FF and MF strikers were associated with shorter horizontal distance from the heel of the support side to the COM at touchdown, greater maximum knee flexion velocity during the swing-leg phase, and higher maximum hip extension velocity during the support-leg phase. In summary, our results suggest that forefoot or midfoot strike are likely useful to improve step frequency and sprint speed in preadolescent boys rather than rearfoot strike. The current findings will be useful for understanding the characteristics of the development of sprinting performance in preadolescent children.

## Supporting information

S1 Fig Illustration of different foot strike patterns noted in this study. Participants who contacted the ground with the rearfoot were included in the RF group (n = 12), whereas those who contacted the ground with the forefoot or midfoot were included in the FF/MF group (n = 12).
(DOCX)

S2 Fig Definition of kinematic variables describing sprint motion. (DOCX)

S1 Table Body height and body mass statistics of the study participants. (DOCX)

S2 Table Descriptive data regarding sprint performance in the 50-m sprint test. The *p* value shows the result of the t-test.
(DOCX)

S3 Table Pearson’s correlation matrix among spatiotemporal variables. (DOCX)

S4 Table Results of ANCOVA in the spatiotemporal variables between the groups with sprint speed as the covariate. (DOCX)

S5 Table Descriptive data for each group in kinematics variables. The *p* value shows the result of the t-test.
(DOCX)

S1 File Minimal data set. (DOCX)
