# Supplementary material for: Differences in sprinting performance and kinematics between preadolescent boys who are fore/mid and rear foot strikers
Source: PLoS One. 2018 Oct 18;13(10):e0205906. doi: 10.1371/journal.pone.0205906 (PMC6193701; doi:10.1371/journal.pone.0205906)
Supplement: S1 Fig — Participants who contacted the ground with the rearfoot were included in the RF group (n = 12), whereas those who contacted the ground with the forefoot or midfoot were included in the FF/MF group (n = 12). (DOCX) [file pone.0205906.s001.docx]

**S1 Fig. Illustration of different foot strike patterns noted in this study.**

**
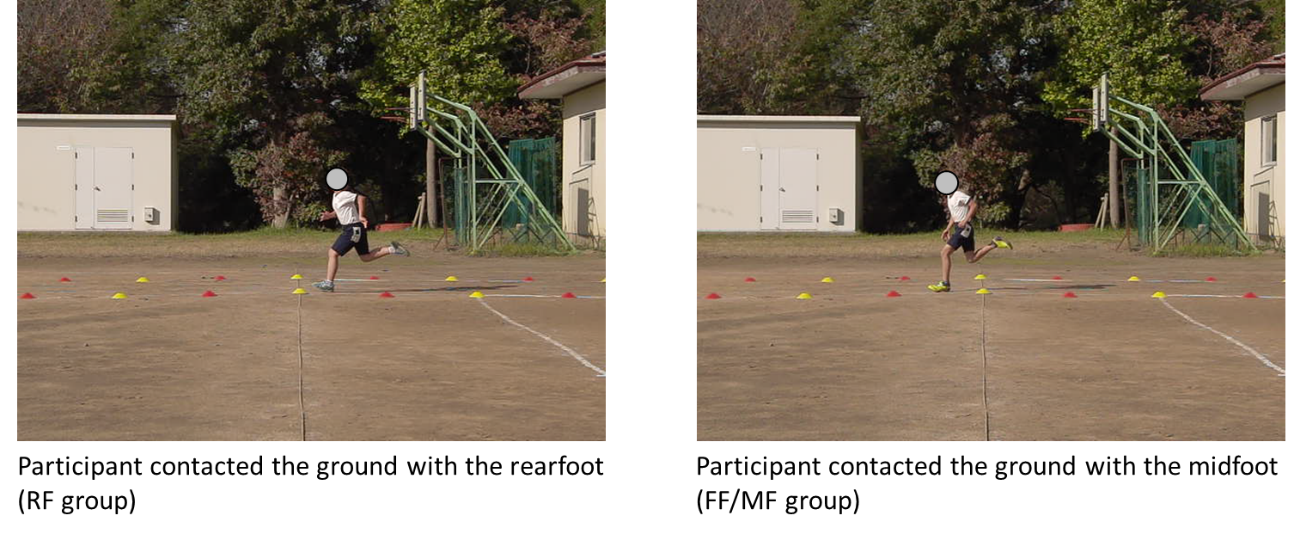
**

Participants who contacted the ground with the rearfoot were included in the RF group (n=12), whereas those who contacted the ground with the forefoot or midfoot were included in the FF/MF group (n=12).
