# Supplementary figures and images for: Differences in sprinting performance and kinematics between preadolescent boys who are fore/mid and rear foot strikers
Source: PLoS One. 2018 Oct 18;13(10):e0205906. doi: 10.1371/journal.pone.0205906 (PMC6193701; doi:10.1371/journal.pone.0205906)

**S2 Fig. Definition of kinematic variables describing sprint motion. .**


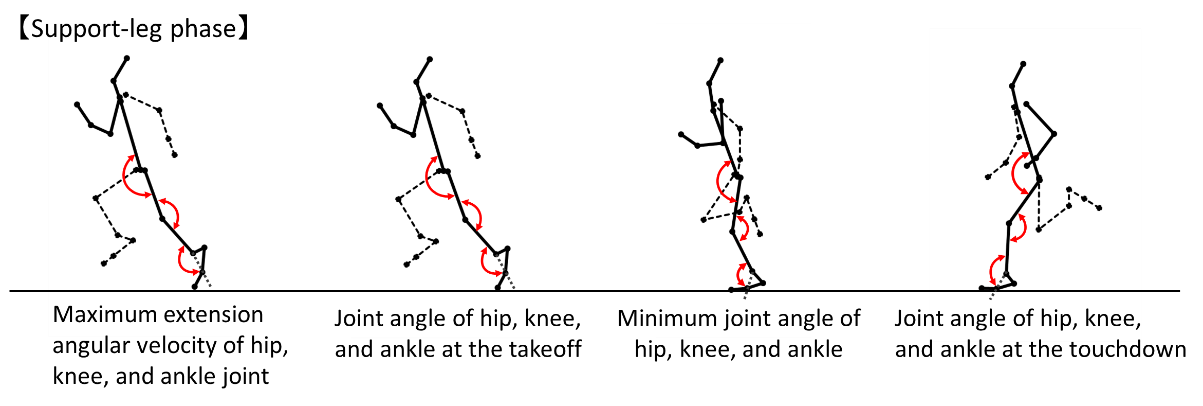


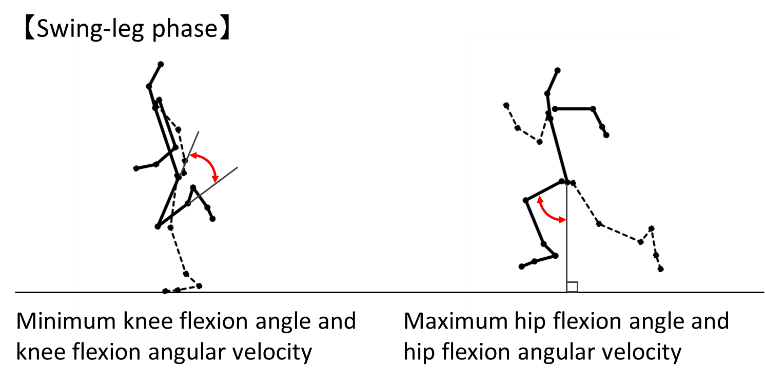

Supplement: S2 Fig — (DOCX) [file pone.0205906.s002.docx]
