# Supplementary material for: Differences in sprinting performance and kinematics between preadolescent boys who are fore/mid and rear foot strikers
Source: PLoS One. 2018 Oct 18;13(10):e0205906. doi: 10.1371/journal.pone.0205906 (PMC6193701; doi:10.1371/journal.pone.0205906)
Supplement: S1 Table — (DOCX) [file pone.0205906.s003.docx]

**S1 Table. Body height and body mass statistics of the study participants.**

| Characteristic | RF group (n=12) | FF/MF group (n=12) | *p* |
| --- | --- | --- | --- |
| Body height (m) | 1.42 ± 0.06 | 1.38 ± 0.08 | 0.242 |
| Body mass (kg) | 37.8 ± 7.4 | 33.1 ± 5.6 | 0.094 |
