# Supplementary material for: Differences in sprinting performance and kinematics between preadolescent boys who are fore/mid and rear foot strikers
Source: PLoS One. 2018 Oct 18;13(10):e0205906. doi: 10.1371/journal.pone.0205906 (PMC6193701; doi:10.1371/journal.pone.0205906)
Supplement: S2 Table — The p value shows the result of the t-test. (DOCX) [file pone.0205906.s004.docx]

**S2 Table. Descriptive data regarding sprint performance in the 50-m sprint test.**

| Variable | RF group (n=12) | FF/MF group (n=12) | p |
| --- | --- | --- | --- |
| Time on the 50-m sprint test (s) | 9.63 ± 0.51 | 9.08 ± 0.52 | 0.017 |
| Sprint speed (m/s) | 6.08 ± 0.40 | 6.53 ± 0.46 | 0.017 |
| Step length (m) | 1.58 ± 0.14 | 1.53 ± 0.11 | 0.303 |
| Step frequency (step/s) | 3.86 ± 0.22 | 4.29 ± 0.28 | 0.001 |
| Foot contact time (s) | 0.160 ± 0.013 | 0.132 ± 0.013 | 0.001 |
| Aerial time (s) | 0.108 ± 0.022 | 0.099 ± 0.012 | 0.180 |

The *p* value shows the result of the t*-*test.
