# Supplementary material for: Differences in sprinting performance and kinematics between preadolescent boys who are fore/mid and rear foot strikers
Source: PLoS One. 2018 Oct 18;13(10):e0205906. doi: 10.1371/journal.pone.0205906 (PMC6193701; doi:10.1371/journal.pone.0205906)
Supplement: S3 Table — (DOCX) [file pone.0205906.s005.docx]

**S3 Table . Pearson's correlation matrix among spatiotemporal variables**

| Variable |  | Sprint speed | Step length | Step frequency | Foot contact time | Aerial time |
| --- | --- | --- | --- | --- | --- | --- |
| Time on the 50-m sprint test | r | -.966 | -.446 | -.493 | .607 | 0.089 |
|  | *p* | 0.001 | 0.029 | 0.014 | 0.002 | 0.679 |
| Sprint speed | r |  | .460 | .507 | -.603 | -.085 |
|  | *p* |  | 0.024 | 0.011 | 0.002 | 0.693 |
| Step length | r |  |  | -.529 | .139 | .595 |
|  | *p* |  |  | 0.008 | 0.518 | 0.002 |
| Step frequency | r |  |  |  | -.705 | -.662 |
|  | *p* |  |  |  | 0.001 | 0.001 |
| Foot contact time | r |  |  |  |  | .112 |
|  | *p* |  |  |  |  | 0.601 |
