# Supplementary material for: Differences in sprinting performance and kinematics between preadolescent boys who are fore/mid and rear foot strikers
Source: PLoS One. 2018 Oct 18;13(10):e0205906. doi: 10.1371/journal.pone.0205906 (PMC6193701; doi:10.1371/journal.pone.0205906)
Supplement: S4 Table — (DOCX) [file pone.0205906.s006.docx]

**S4 Table. Results of ANCOVA in the spatiotemporal variables between the groups with sprint speed as the covariate**

| Source | | Sum of Squares | df | Mean Square | F | *p* |
| --- | --- | --- | --- | --- | --- | --- |
| Sprint speed | Step length | 0.143 | 1 | 0.143 | 16.394 | 0.001 |
|  | Step frequency | 0.115 | 1 | 0.115 | 1.879 | 0.185 |
|  | Foot contact time | 0.001 | 1 | 0.001 | 4.444 | 0.047 |
|  | Aerial time | 2.516E-05 | 1 | 2.516E-05 | 0.080 | 0.780 |
| Group | Step length | 0.087 | 1 | 0.087 | 9.957 | 0.005 |
|  | Step frequency | 0.569 | 1 | 0.569 | 9.272 | 0.006 |
|  | Foot contact time | 0.002 | 1 | 0.002 | 15.556 | 0.001 |
|  | Aerial time | 0.001 | 1 | 0.001 | 1.756 | 0.199 |
| Error | Step length | 0.184 | 21 | 0.009 |  |  |
|  | Step frequency | 1.288 | 21 | 0.061 |  |  |
|  | Foot contact time | 0.003 | 21 | 0.000 |  |  |
|  | Aerial time | 0.007 | 21 | 0.000 |  |  |
