# Supplementary material for: Differences in sprinting performance and kinematics between preadolescent boys who are fore/mid and rear foot strikers
Source: PLoS One. 2018 Oct 18;13(10):e0205906. doi: 10.1371/journal.pone.0205906 (PMC6193701; doi:10.1371/journal.pone.0205906)
Supplement: S5 Table — The p value shows the result of the t-test. (DOCX) [file pone.0205906.s007.docx]

**S5 Table. Descriptive data for each group in kinematics variables.**

| Variable | | RF group (n=12) | FF/MF group (n=12) | *p* |
| --- | --- | --- | --- | --- |
| Support-leg phase | |  |  |  |
| Joint angle at touchdown (degree) | Hip | 116.7 ± 7.9 | 118.1 ± 8.9 | 0.673 |
|  | Knee | 142.0 ± 3.9 | 139.3 ± 7.2 | 0.354 |
|  | Ankle | 120.9 ± 7.6 | 126.4 ± 8.8 | 0.115 |
| Minimum joint angle  (degree) | Hip | 116.7 ± 7.9 | 118.1 ± 8.9 | 0.673 |
|  | Knee | 123.5 ± 7.4 | 125.3 ± 7.3 | 0.566 |
|  | Ankle | 108.0 ± 9.6 | 115.8 ± 9.5 | 0.057 |
| Joint angle at takeoff  (degree) | Hip | 175.4 ± 3.5 | 175.2 ± 4.0 | 0.887 |
|  | Knee | 147.7 ± 5.6 | 148.5 ± 6.2 | 0.770 |
|  | Ankle | 147.7 ± 7.8 | 151.1 ± 6.0 | 0.247 |
| ROM | Hip extension | 51.9 ± 6.3 | 50.0 ± 7.8 | 0.505 |
|  | Knee flexion | -18.5 ± 4.2 | -14.1 ± 4.2 | 0.017 |
|  | Knee extension | 24.2 ± 4.3 | 23.2 ± 4.7 | 0.578 |
|  | Ankle flexion | -13.0 ± 6.5 | -10.6 ± 5.8 | 0.364 |
|  | Ankle extension | 39.7 ± 6.4 | 35.3 ± 7.8 | 0.136 |
| Maximum extension velocity (degree/s) | Hip | 686.7 ± 115.7 | 793.9 ± 98.1 | 0.023 |
|  | Knee | 490.1 ± 83.3 | 536.4 ± 85.0 | 0.191 |
|  | Ankle | 707.8 ± 145.4 | 707.9 ± 149.8 | 0.999 |
| Swing-leg phase | |  |  |  |
| Maximum hip flexion angle (degree) | | 57.9 ± 5.8 | 61.6 ± 6.2 | 0.151 |
| Maximum hip flexion velocity (degree/s) | | 616.9 ± 84.3 | 672.9 ± 75.0 | 0.100 |
| Minimum knee flexion angle (degree) | | 41.8 ± 13.5 | 39.3 ± 12.4 | 0.644 |
| Maximum knee flexion velocity (degree/s) | | -932.4 ± 112.3 | -1026.0 ± 106.8 | 0.048 |
| COM trajectory | |  |  |  |
| The COM height (cm) | At lowest point | 79.6 ± 4.0 | 77.1 ± 4.6 | 0.170 |
|  | At highest point | 86.0 ± 4.5 | 82.3 ± 4.8 | 0.068 |
|  | At touchdown | 81.9 ± 4.4 | 78.9 ± 4.8 | 0.122 |
|  | At takeoff | 85.2 ± 4.2 | 81.7 ± 4.8 | 0.072 |
| Vertical displacement (cm) | | 6.4 ± 1.2 | 5.2 ± 1.0 | 0.017 |
| Horizontal displacement  (cm) | For the Support | 95.6 ± 8.0 | 85.7 ± 7.5 | 0.005 |
|  | For the Aerial | 67.2 ± 13.8 | 66.4 ± 9.8 | 0.873 |
| Horizontal distance from the heel to the COM at touchdown (cm) | 15.3 ± 3.8 | 9.3 ± 3.1 | 0.001 |  |

The *p* value shows the result of the t*-*test.
