# Supplementary material for: Differences in sprinting performance and kinematics between preadolescent boys who are fore/mid and rear foot strikers
Source: PLoS One. 2018 Oct 18;13(10):e0205906. doi: 10.1371/journal.pone.0205906 (PMC6193701; doi:10.1371/journal.pone.0205906)
Supplement: S1 File — (DOCX) [file pone.0205906.s008.docx]

**S1 File. Minimal data set.docx**

Descriptive data for each group

| Variable | group | df | Average | SD | SE |
| --- | --- | --- | --- | --- | --- |
| Body height (m) | FF/MF | 12 | 1.3848 | 0.07502 | 0.02166 |
|  | RF | 12 | 1.4181 | 0.05998 | 0.01732 |
| Body mass (kg) | FF/MF | 12 | 33.1333 | 5.57027 | 1.60800 |
|  | RF | 12 | 37.8167 | 7.42586 | 2.14366 |
| Time on the 50-m sprint test (s) | FF/MF | 12 | 9.0842 | 0.51546 | 0.14880 |
|  | RF | 12 | 9.6258 | 0.51511 | 0.14870 |
| Sprint speed (m/s) | FF/MF | 12 | 6.5325 | 0.45464 | 0.13124 |
|  | RF | 12 | 6.0792 | 0.40500 | 0.11691 |
| Step length (m) | FF/MF | 12 | 1.5258 | 0.10833 | 0.03127 |
|  | RF | 12 | 1.5783 | 0.13422 | 0.03875 |
| Step frequency (step/s) | FF/MF | 12 | 4.2892 | 0.27750 | 0.08011 |
|  | RF | 12 | 3.8617 | 0.22478 | 0.06489 |
| Foot contact time (s) | FF/MF | 12 | 0.1325 | 0.01266 | 0.00365 |
|  | RF | 12 | 0.1603 | 0.01316 | 0.00380 |
| Aerial time (s) | FF/MF | 12 | 0.0985 | 0.01173 | 0.00339 |
|  | RF | 12 | 0.1083 | 0.02159 | 0.00623 |
| Hip joint angle at touchdown (degree) | FF/MF | 12 | 118.1417 | 8.90745 | 2.57136 |
|  | RF | 12 | 116.6583 | 7.92757 | 2.28849 |
| Knee joint angle at touchdown (degree) | FF/MF | 12 | 139.3250 | 7.15111 | 2.06435 |
|  | RF | 12 | 142.0417 | 6.88232 | 1.98675 |
| Ankle joint angle at touchdown (degree) | FF/MF | 12 | 126.4500 | 8.80191 | 2.54089 |
|  | RF | 12 | 120.9250 | 7.58421 | 2.18937 |
| Minimum hip joint angle in the support-leg phase (degree) | FF/MF | 12 | 118.1417 | 8.90745 | 2.57136 |
|  | RF | 12 | 116.6583 | 7.92757 | 2.28849 |
| Minimum knee joint angle in the support-leg phase (degree) | FF/MF | 12 | 125.2667 | 7.30720 | 2.10941 |
|  | RF | 12 | 123.5167 | 7.37118 | 2.12788 |
| Minimum ankle joint angle in the support-leg phase (degree) | FF/MF | 12 | 115.7917 | 9.49980 | 2.74236 |
|  | RF | 12 | 107.9667 | 9.58970 | 2.76831 |
| Hip joint angle at takeoff (degree) | FF/MF | 12 | 175.2167 | 3.98881 | 1.15147 |
|  | RF | 12 | 175.4500 | 3.47890 | 1.00427 |
| Knee joint angle at takeoff (degree) | FF/MF | 12 | 148.4500 | 6.24784 | 1.80359 |
|  | RF | 12 | 147.7250 | 5.57219 | 1.60855 |
| Ankle joint angle at takeoff (degree) | FF/MF | 12 | 151.0750 | 5.96583 | 1.72219 |
|  | RF | 12 | 147.7083 | 7.73275 | 2.23225 |
| Hip extension ROM during the support-leg phase (degree) | FF/MF | 12 | 49.9583 | 7.84897 | 2.26580 |
|  | RF | 12 | 51.9250 | 6.27435 | 1.81125 |
| Knee extension ROM during the support-leg phase (degree) | FF/MF | 12 | -14.0000 | 4.39007 | 1.26730 |
|  | RF | 12 | -18.5833 | 4.10007 | 1.18359 |
| Knee flexion ROM during the support-leg phase (degree) | FF/MF | 12 | 23.1833 | 4.65985 | 1.34518 |
|  | RF | 12 | 24.2083 | 4.27242 | 1.23334 |
| Ankle extension ROM during the support-leg phase (degree) | FF/MF | 12 | -10.5833 | 5.77547 | 1.66723 |
|  | RF | 12 | -13.0833 | 6.48717 | 1.87268 |
| Ankle flexion ROM during the support-leg phase (degree) | FF/MF | 12 | 35.2667 | 7.77272 | 2.24379 |
|  | RF | 12 | 39.7333 | 6.35643 | 1.83494 |
| Maximum hip extension velocity (degree/s) | FF/MF | 12 | 793.9250 | 98.14558 | 28.33219 |
|  | RF | 12 | 686.7417 | 115.70103 | 33.40001 |
| Maximum knee extension velocity (degree/s) | FF/MF | 12 | 536.4167 | 85.00309 | 24.53828 |
|  | RF | 12 | 490.0833 | 83.29792 | 24.04604 |
| Maximum ankle extension velocity (degree/s) | FF/MF | 12 | 707.8917 | 149.73216 | 43.22395 |
|  | RF | 12 | 707.8250 | 145.40263 | 41.97412 |
| Maximum hip flexion angle in the swing-leg phase (degree) | FF/MF | 12 | 61.5917 | 6.21851 | 1.79513 |
|  | RF | 12 | 57.9583 | 5.76675 | 1.66472 |
| Maximum hip flexion velocity in the swing-leg phase (degree/s) | FF/MF | 12 | 672.9000 | 74.95057 | 21.63636 |
|  | RF | 12 | 616.8917 | 84.30596 | 24.33704 |
| Minimum knee flexion angle in the swing-leg phase (degree) | FF/MF | 12 | 39.2833 | 12.43111 | 3.58855 |
|  | RF | 12 | 41.7583 | 13.50350 | 3.89812 |
| Maximum knee flexion velocity in the swing-leg phase (degree/s) | FF/MF | 12 | -1026.0000 | 106.81845 | 30.83583 |
|  | RF | 12 | -932.4167 | 112.24442 | 32.40217 |
| The COM height at lowest point (cm) | FF/MF | 12 | 77.0929 | 4.58122 | 1.32248 |
|  | RF | 12 | 79.5868 | 4.01450 | 1.15889 |
| The COM height at highest point (cm) | FF/MF | 12 | 82.3022 | 4.82163 | 1.39188 |
|  | RF | 12 | 85.9686 | 4.54196 | 1.31115 |
| The COM height at touchdown (cm) | FF/MF | 12 | 78.9172 | 4.83186 | 1.39484 |
|  | RF | 12 | 81.9392 | 4.35079 | 1.25596 |
| The COM height at takeoff (cm) | FF/MF | 12 | 81.7168 | 4.82349 | 1.39242 |
|  | RF | 12 | 85.2121 | 4.23258 | 1.22184 |
| Vertical COM displacement (cm) | FF/MF | 12 | 5.2092 | 0.95556 | 0.27585 |
|  | RF | 12 | 6.3818 | 1.24830 | 0.36035 |
| Horizontal COM displacement for the Support (cm) | FF/MF | 12 | 85.6566 | 7.51369 | 2.16902 |
|  | RF | 12 | 95.6372 | 7.96522 | 2.29936 |
| Horizontal COM displacement for the Aerial (cm) | FF/MF | 12 | 66.4055 | 9.75910 | 2.81721 |
|  | RF | 12 | 67.1948 | 13.78811 | 3.98029 |
| Horizontal distance from the heel to the COM at touchdown (cm) | FF/MF | 12 | 9.2616 | 3.13665 | 0.90547 |
|  | RF | 12 | 15.3270 | 3.84977 | 1.11133 |

Data of t-test for comparing 2 groups

| Variable | F value | *t* value | df | P value |
| --- | --- | --- | --- | --- |
| Body height (m) | 0.139 | -1.202 | 22 | 0.242 |
| Body mass (kg) | 0.519 | -1.748 | 22 | 0.094 |
| Time on the 50-m sprint test (s) | 0.021 | -2.575 | 22 | 0.017 |
| Sprint speed (m/s) | 0.103 | 2.579 | 22 | 0.017 |
| Step length (m) | 0.612 | -1.054 | 22 | 0.303 |
| Step frequency (step/s) | 1.060 | 4.147 | 22 | 0.000 |
| Foot contact time (s) | 0.015 | -5.263 | 22 | 0.000 |
| Aerial time (s) | 2.826 | -1.386 | 22 | 0.180 |
| Hip joint angle at touchdown (degree) | 0.623 | 0.431 | 22 | 0.671 |
| Knee joint angle at touchdown (degree) | 0.112 | -0.948 | 22 | 0.353 |
| Ankle joint angle at touchdown (degree) | 0.020 | 1.647 | 22 | 0.114 |
| Minimum hip joint angle in the support-leg phase (degree) | 0.623 | 0.431 | 22 | 0.671 |
| Minimum knee joint angle in the support-leg phase (degree) | 0.024 | 0.584 | 22 | 0.565 |
| Minimum ankle joint angle in the support-leg phase (degree) | 0.183 | 2.008 | 22 | 0.057 |
| Hip joint angle at takeoff (degree) | 1.057 | -0.153 | 22 | 0.880 |
| Knee joint angle at takeoff (degree) | 0.145 | 0.300 | 22 | 0.767 |
| Ankle joint angle at takeoff (degree) | 0.193 | 1.194 | 22 | 0.245 |
| Hip extension ROM during the support-leg phase (degree) | 1.181 | -0.678 | 22 | 0.505 |
| Knee extension ROM during the support-leg phase (degree) | 0.030 | 2.643 | 22 | 0.015 |
| Knee flexion ROM during the support-leg phase (degree) | 0.598 | -0.562 | 22 | 0.580 |
| Ankle extension ROM during the support-leg phase (degree) | 0.088 | 0.997 | 22 | 0.330 |
| Ankle flexion ROM during the support-leg phase (degree) | 0.001 | -1.541 | 22 | 0.138 |
| Maximum hip extension velocity (degree/s) | 0.198 | 2.447 | 22 | 0.023 |
| Maximum knee extension velocity (degree/s) | 0.012 | 1.349 | 22 | 0.191 |
| Maximum ankle extension velocity (degree/s) | 0.011 | 0.001 | 22 | 0.999 |
| Maximum hip flexion angle in the swing-leg phase (degree) | 0.426 | 1.484 | 22 | 0.152 |
| Maximum hip flexion velocity in the swing-leg phase (degree/s) | 0.124 | 1.720 | 22 | 0.099 |
| Minimum knee flexion angle in the swing-leg phase (degree) | 0.006 | -0.467 | 22 | 0.645 |
| Maximum knee flexion velocity in the swing-leg phase (degree/s) | 0.048 | -2.092 | 22 | 0.048 |
| The COM height at lowest point (cm) | 0.498 | -1.418 | 22 | 0.170 |
| The COM height at highest point (cm) | 0.305 | -1.917 | 22 | 0.068 |
| The COM height at touchdown (cm) | 0.357 | -1.610 | 22 | 0.122 |
| The COM height at takeoff (cm) | 0.584 | -1.887 | 22 | 0.072 |
| Vertical COM displacement (cm) | 1.292 | -2.584 | 22 | 0.017 |
| Horizontal COM displacement for the Support (cm) | 0.821 | -3.157 | 22 | 0.005 |
| Horizontal COM displacement for the Aerial (cm) | 1.909 | -0.162 | 22 | 0.873 |
| Horizontal distance from the heel to the COM at touchdown (cm) | 0.130 | -4.231 | 22 | 0.000 |

Pearson's correlation matrix among spatiotemporal variables

| Variable |  | Sprint speed | Step length | Step frequency | Foot contact time | Aerial time |
| --- | --- | --- | --- | --- | --- | --- |
| Time on the 50-m sprint test | r | -.966 | -.446 | -.493 | .607 | 0.089 |
|  | *p* | 0.001 | 0.029 | 0.014 | 0.002 | 0.679 |
| Sprint speed | r |  | .460 | .507 | -.603 | -.085 |
|  | *p* |  | 0.024 | 0.011 | 0.002 | 0.693 |
| Step length | r |  |  | -.529 | .139 | .595 |
|  | *p* |  |  | 0.008 | 0.518 | 0.002 |
| Step frequency | r |  |  |  | -.705 | -.662 |
|  | *p* |  |  |  | 0.001 | 0.001 |
| Foot contact time | r |  |  |  |  | .112 |
|  | *p* |  |  |  |  | 0.601 |

Results of ANCOVA in the spatiotemporal variables between the groups with sprint speed as the covariate

| Source | | Sum of Squares | df | Mean Square | F | *p* |
| --- | --- | --- | --- | --- | --- | --- |
| Sprint speed | Step length | 0.143 | 1 | 0.143 | 16.394 | 0.001 |
|  | Step frequency | 0.115 | 1 | 0.115 | 1.879 | 0.185 |
|  | Foot contact time | 0.001 | 1 | 0.001 | 4.444 | 0.047 |
|  | Aerial time | 2.516E-05 | 1 | 2.516E-05 | 0.080 | 0.780 |
| Group | Step length | 0.087 | 1 | 0.087 | 9.957 | 0.005 |
|  | Step frequency | 0.569 | 1 | 0.569 | 9.272 | 0.006 |
|  | Foot contact time | 0.002 | 1 | 0.002 | 15.556 | 0.001 |
|  | Aerial time | 0.001 | 1 | 0.001 | 1.756 | 0.199 |
| Error | Step length | 0.184 | 21 | 0.009 |  |  |
|  | Step frequency | 1.288 | 21 | 0.061 |  |  |
|  | Foot contact time | 0.003 | 21 | 0.000 |  |  |
|  | Aerial time | 0.007 | 21 | 0.000 |  |  |
